# Cardiolipin dynamics and binding to conserved residues in the mitochondrial ADP/ATP carrier

**DOI:** 10.1016/j.bbamem.2018.01.017

**Published:** 2018-05

**Authors:** Anna L. Duncan, Jonathan J. Ruprecht, Edmund R.S. Kunji, Alan J. Robinson

**Affiliations:** Medical Research Council, Mitochondrial Biology Unit, Cambridge Biomedical Campus, Cambridge CB2 0XY, UK

**Keywords:** AAC, ADP/ATP carrier, CDL, tetraoleoyl cardiolipin, POPC, 1-palmitoyl-2-oleoyl-*sn*-glycero-3-phosphatidylcholine, POPE, 1-palmitoyl-2-oleoyl-*sn*-glycero-3-phosphatidylethanolamine, TM-Lys, trimethyllysine, Mitochondria, Trimethyllysine, Cardiolipin, Molecular dynamics simulation, Adenine nucleotide translocator, Adenine nucleotide transporter

## Abstract

Cardiolipin in eukaryotes is found in the mitochondrial inner membrane, where it interacts with membrane proteins and, although not essential, is necessary for the optimal activity of a number of proteins. One of them is the mitochondrial ADP/ATP carrier, which imports ADP into the mitochondrion and exports ATP. In the crystal structures, cardiolipin is bound to three equivalent sites of the ADP/ATP carrier, but its role is unresolved. Conservation of residues at these cardiolipin binding sites across other members of the mitochondrial carrier superfamily indicates cardiolipin binding is likely to be important for the function of all mitochondrial carriers. Multiscale simulations were performed in a cardiolipin-containing membrane to investigate the dynamics of cardiolipin around the yeast and bovine ADP/ATP carriers in a lipid bilayer and the properties of the cardiolipin-binding sites. In coarse-grain simulations, cardiolipin molecules bound to the carriers for longer periods of time than phosphatidylcholine and phosphatidylethanolamine lipids—with timescales in the tens of microseconds. Three long-lived cardiolipin binding sites overlapped with those in the crystal structures of the carriers. Other shorter-lived cardiolipin interaction sites were identified in both membrane leaflets. However, the timescales of the interactions were of the same order as phosphatidylcholine and phosphatidylethanolamine, suggesting that these sites are not specific for cardiolipin binding. The calculation of lipid binding times and the overlap of the cardiolipin binding sites between the structures and simulations demonstrate the potential of multiscale simulations to investigate the dynamics and behavior of lipids interacting with membrane proteins.

## Highlights

• Coarse-grained models of AAC in mixed lipid bilayers were simulated.
• Three long-lived cardiolipin sites correspond to those in the crystal structures.
• No other long-lived binding sites were observed for cardiolipin or other phospholipids.
• Trimethylation of Lys-51 of AAC had no effect on cardiolipin interactions.

## Graphical abstract

**Figure:**
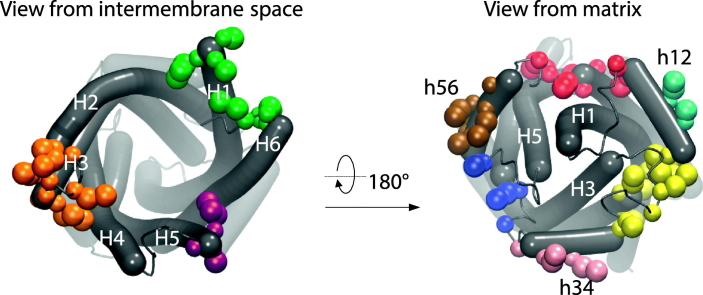

## 1 Introduction

Cardiolipin is an anionic lipid of the mitochondrial inner membrane of eukaryotic cells, and the plasma membranes of bacteria. Most is in the inner leaflet [1,2], at or near its site of synthesis [3], and it has an unusual structure, with two linked phosphatidyl groups giving the lipid four acyl chains and a double negative charge [4,5]. Cardiolipin associates with many mitochondrial membrane proteins, notably of the respiratory chain. Although not essential in yeast, cardiolipin is required for optimal activity of mitochondria [6], and in metazoans, cardiolipin may be required: deletion of an enzyme required for cardiolipin biosynthesis is embryonically lethal [7]. Various roles have been proposed for cardiolipin, including as a proton buffer and for increasing membrane curvature, as well as a chaperone promoting protein folding, oligomerisation and formation of membrane domains with supercomplexes [6]. Cardiolipin deficiency adversely affects mitochondrial function, particularly the membrane-bound proteins involved in oxidative phosphorylation [8], and diseases of dysfunctional cardiolipin metabolism underline the importance of understanding the role of cardiolipin [9]. For example, Barth Syndrome—a rare disease with symptoms including cardiomyopathy and muscle weakness—is caused by mutations in the tafazzin gene that encodes an enzyme in the cardiolipin biosynthetic pathway [10]. Abnormal cardiolipin content is also linked to mitochondrial dysfunction in cancer [11], neurodegenerative disease [12], and aging [13].

Cardiolipin associates strongly with the mitochondrial ADP/ATP carriers (AAC) in a ratio of 3:1, as evidenced by ^31^P NMR and ESR spectroscopic measurements [14,15], and by its presence in crystal structures despite stringent washes during protein purification [[16], [17], [18]]. Although cardiolipin is not essential for AAC function, it promotes the stability of the AAC [19], improves transport activity of the AACs of yeast [8,20] and metazoa [21], and may facilitate conformational change during ADP/ATP translocation [17].

The AACs are members of the mitochondrial carrier superfamily [22], and facilitate the one-to-one exchange of ADP and ATP across the inner mitochondrial membrane [23] as a key component of oxidative phosphorylation. They are monomeric proteins with six transmembrane helices formed from three homologous domains [16,24,25], with the two transmembrane helices of each domain linked on the mitochondrial matrix side by a short matrix helix: h12, h34 and h56. The consensus binding site for the cardiolipin headgroup, determined from the crystal structures, is between the matrix and even-numbered helices at the interfaces of the three domains [[16], [17], [18]]. In the binding site, the cardiolipin headgroups are close to two conserved motifs —a [YWF][KR]G motif at the N-terminus of the even-numbered transmembrane helices and a [YF]xG motif at the N-terminus of the matrix helices—and interact with their positively charged helix dipoles and amide groups [17].

Lys-51, which belongs to the [YWF][KR]G motif, is trimethylated in the bovine AAC [26], but its role is uninvestigated. In general, the role of lysine trimethylation in mitochondrial proteins is unclear, but cardiolipin binding to the c-ring of ATP synthase may be promoted by trimethylation of a lysine residue in the lipid headgroup region [27], although previous coarse-grained simulations of the trimethylated c-ring of the bovine ATP synthase showed that overall, cardiolipin–c-ring interactions are not significantly altered by trimethylation [28]. Trimethylated lysine (TM-Lys) retains the positive charge of lysine, although the replacement of hydrogen atoms by methyl groups leads to a group that is larger, more hydrophobic, more polarisable and without hydrogen-bonding ability—similar to a choline headgroup in phosphatidylcholine phospholipids. The trimethyllysines will attract the negatively charged cardiolipin, and so in the c-ring may mark sites that favor cardiolipin binding over other phospholipids [27]. Further, TM-Lys may form only electrostatic interactions with negatively charged ligands, perhaps promoting shorter-lived, but specific, interactions.

Cardiolipin binding sites have been resolved in crystal structures of mitochondrial membrane proteins, but they provide a static view in the context of a detergent micelle rather than a membrane. Previously, we used molecular dynamics simulations to identify and characterize conserved binding sites selective for cardiolipin in the c-rings of ATP synthase that are unresolved in crystal structures [28]. The longer timescales of coarse-grained simulations (compared to atomistic simulations) make them particularly suitable for studying protein–lipid interactions that occur on the microsecond time-scale, and combining coarse-grained and atomistic simulation allows study of both atomistic detail and long-term protein–lipid interaction dynamics [[28], [29], [30], [31], [32], [33], [34], [35]]. Thus, to investigate the dynamics of AAC–cardiolipin interactions and their possible functional roles, multiscale simulations were run of the bovine AAC1 with and without trimethylated Lys-51 (TM-Lys-51), and the *S. cerevisiae* AAC2 and AAC3 isoforms, embedded in hydrated lipid membranes containing 1-palmitoyl-2-oleoyl-*sn*-glycero-3-phosphocholine (POPC), 1-palmitoyl-2-oleoyl-*sn*-glycero-3-phosphoethanolamine (POPE) and tetraoleoyl cardiolipin. The objectives of these studies were to: (i) evaluate whether simulations replicate the cardiolipin binding poses of the crystal structures, (ii) determine if cardiolipin behaves differently to other phospholipids around the AAC, (iii) identify any additional cardiolipin binding sites, and (iv) evaluate whether trimethylation of Lys-51 in the bovine AAC affects cardiolipin binding. In these simulations, cardiolipin interactions with the AAC were significantly more long-lived than POPC and POPE interactions. Three equivalent cardiolipin binding sites—one in each of the three domains of the carrier—had particularly long-lived interactions, and these sites overlapped with the three binding sites in the crystal structures and with previously published simulation results [34]. Other, novel cardiolipin interaction sites were identified, but these had much more short-lived interactions with cardiolipin. Lys-51 trimethylation had little effect on cardiolipin interaction with the bovine AAC. Although some specific interactions of the cardiolipin binding sites vary between the crystal structure and simulations, the results provide insight into the cardiolipin dynamics at the surface of the AAC and demonstrate the efficacy of coarse-grained simulations in identifying cardiolipin-binding sites.

## 2 Results

### 2.1 Binding sites of cardiolipin in the structures of the yeast and bovine AAC

**Fig. 1:**
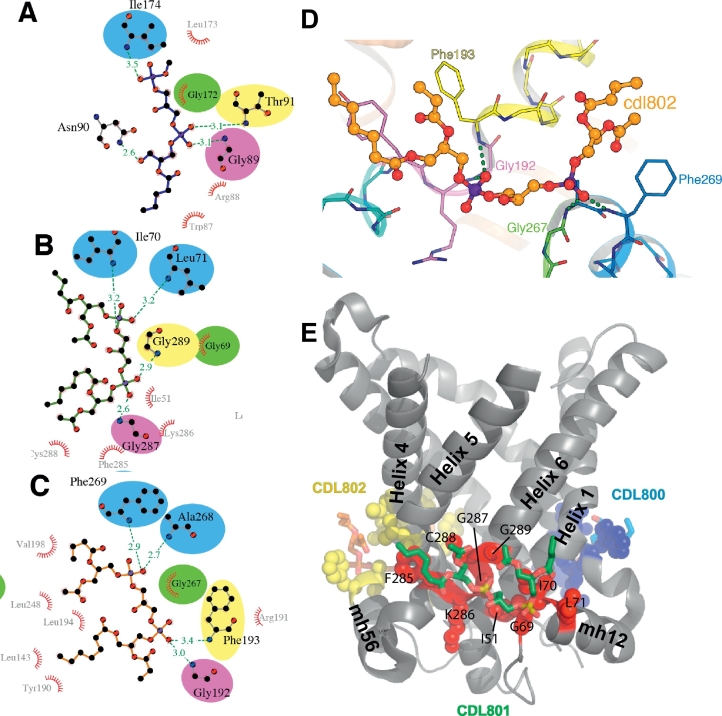
Cardiolipin–protein interactions in the crystal structure of the yeast AAC2. Interactions of yeast AAC2 (PDB ID: 4C9H chain A) with (A) cardiolipin CDL800 (blue), (B) cardiolipin CDL801 (green) and (C) cardiolipin CDL802 (orange). (D) Detailed view of the binding site for cardiolipin, CDL802. In (A–D) interactions are shown: hydrogen bonds (green dashed lines) with their length (Å); residues in hydrophobic contact with cardiolipin (red arcs with spokes radiating towards the atoms they contact). Also in (A–D), amino acids are coloured according to: the N-terminal end of the matrix helices (blue); the N-terminal end of the even-numbered transmembrane helices (yellow); the [YWF][KR]G motif (violet); the [YF]xG motif (green); and the linker helix (cyan). (E) The binding site of cardiolipin CDL801, showing the position relative to the entire structure. Cardiolipin is shown as green sticks for carbon atoms, with oxygen and phosphorous atoms shown in red and brown, respectively. Interacting amino acids for all three cardiolipin molecules in the structure are shown as spheres (red for those interacting with CDL801; blue for CDL800; and yellow for CDL802). In all panels, cardiolipin has truncated acyl chains, since electron density was present only for the acyl chains close to the headgroup.

In the structures of the bovine AAC1 (PDB:1OKC and PDB:2C3E) and the yeast AAC2 and AAC3 (PDB:4C9G, PDB:4C9H, PDB:4C9J and PDB:4C9Q), three cardiolipins are bound between the N-termini of the matrix and even-numbered α-helices (Figs. 1 and S1). The cardiolipin between helix H2 and matrix helices h12 and h34 is labeled as CDL800; between H4 and matrix helices h34 and h56 as CDL802; and between H6 and matrix helices h12 and h56 as CDL801. The headgroup of each cardiolipin is well resolved in most of the structures—making interactions with AAC—but the acyl chains are only resolved close to the headgroup. However, as described previously [17], the cardiolipins CDL800 and CDL802 in the structure of the bovine AAC (PDB:1OKC) have different conformations to those of the other structures. The headgroup of CDL800 folds back on itself, allowing only one phosphate group of the cardiolipin to interact with the protein (via the glycine residue of the [YWF][KR]G motif and the dipole of matrix helix h12). A phosphatidylcholine lipid molecule is modelled close to CDL800, and appears to interact with the dipole and amide groups of matrix helix h34. Only part of CDL802 is modelled (one phosphatidyl moiety), and it is displaced from the position of equivalent groups in the other structures. In contrast, all of the other cardiolipin molecules are bound in similar positions in the structures of the AAC from bovine (PDB:2C3E) and yeast (PDB:4C9G and PDB:4C9Q). One cardiolipin phosphate interacts with the backbone atoms of the glycine residue of the [YWF][KR]G motifs at the N-terminus of the even-numbered transmembrane α-helices and the other cardiolipin phosphate interacts with the backbone atoms of the residues at the N-terminus of the matrix helices, which are part of the [YF]xG motif (Figs. 1 and S1). There are no hydrogen bonds between cardiolipin phosphates and amino acid side chains, although the side chains of Arg-191 in the yeast AAC2 and Arg-180 in the yeast AAC3 are less than 4 Å from the phosphate of CDL802, suggesting a weak electrostatic interaction. Therefore, cardiolipin binding is not mediated by the basic amino acids of the [YWF][KR]G motif, but rather with backbone atoms at the termini of α-helices. In the PDB:2C3E structure of the bovine AAC, parts of the acyl chains are modelled. One pair of the cardiolipin acyl chains are oriented in the direction of its neighboring transmembrane α-helix; whereas the other pair are oriented almost perpendicular to its neighboring transmembrane α-helices. This conformation suggests that each cardiolipin wraps its chains around the carrier, but it is unknown if this arrangement is retained in a lipid bilayer.

### 2.2 Cardiolipin, but not POPE and POPC, bind specifically with the yeast AAC2 and AAC3

To investigate the dynamics of cardiolipin interactions with the AAC, two coarse-grained models were built of the AAC2 and AAC3 of *S. cerevisiae* [17], and inserted into bilayers containing randomly placed tetraoleoyl cardiolipin, POPC and POPE. After equilibration of each system, 50 μs production simulations were run. Even within the equilibration stage, it appeared cardiolipin molecules had become bound to the AAC. The protein structures remained stable during the simulation and the lipid bilayer was in the liquid phase (Figs. S2 and S3).

**Fig. 2:**
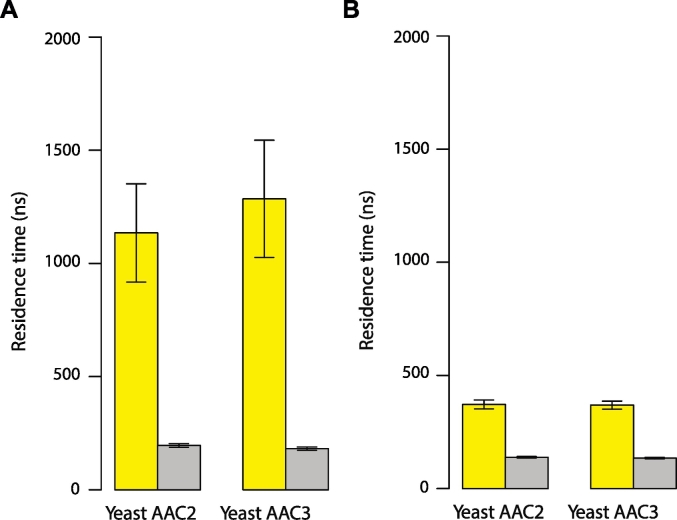
Calculated residence times for cardiolipin (yellow) and phospholipids POPC and POPE (grey) during simulations of yeast AAC2 and AAC3. POPC, POPE and cardiolipin were present in all simulations with a ratio of 9:7:4 (see Materials and methods). Residence times were averaged over the production simulations for the interaction between whole lipids and the entire AAC surface in the (A) inner and (B) outer leaflets.

To compare the lifetime of protein–lipid interactions of cardiolipin with those of POPC and POPE, the residence times of lipids bound to the AAC were calculated from the simulations. The residence time is a parameter extracted from protein–lipid interaction time-correlation decay curves, and provides a measure of the interaction times (i.e. the raw lengths of time for which individual lipids interact with the protein) for an ensemble of lipids. Residence times of cardiolipin were greater than those of POPC and POPE, and cardiolipin in the inner leaflet interacted with AAC2 and AAC3 for longer times than cardiolipin in the outer leaflet (Fig. 2). The residence times of POPC and POPE were similar regardless of the AAC isoform or membrane leaflet (Figs. 2 and S4).

**Fig. 3:**
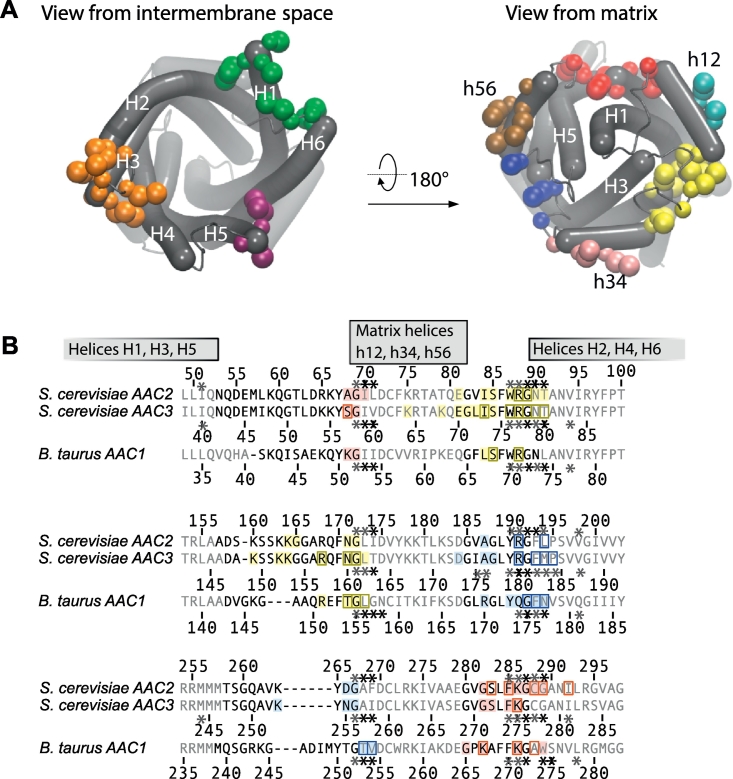
Residues of the yeast AAC2 forming cardiolipin binding sites during coarse-grained simulations. (A) Interacting residues in the outer leaflet (left) and inner leaflet (right), coloured according to binding site. In the inner leaflet amino acids forming binding sites are coloured: CDL800 (yellow); CDL801 (red); CDL802 (blue); h12 (cyan); h34 (pink); h56 (brown). In the outer leaflet, the three identified binding sites are coloured: outer 1 (green); outer 2 (purple); and outer 3 (orange). Protein α-helices (grey tubes) were drawn using Bendix [36]. (B) Protein sequence alignment of yeast AAC2 and AAC3, and bovine AAC1 showing location and conservation of the cardiolipin binding sites: CDL800, CDL801 and CDL802. During coarse-grained and atomistic simulations, cardiolipin binding sites were identified by clustering residues that interacted simultaneously with a single cardiolipin headgroup (see Figs. S5 and S6). Identified interaction sites are coloured in the alignment: site CDL800 (yellow); site CDL801 (red); and site CDL802 (blue). Residues identified during coarse-grained simulations are shown as solid coloured boxes; residues identified in atomistic simulations are shown by coloured outlines. In order to show the overlap with crystal structure data, residues that interact with cardiolipin in crystal structures [17] are highlighted in the alignment with an asterisk (see also Figs. 1 and S1).

**Fig. 4:**
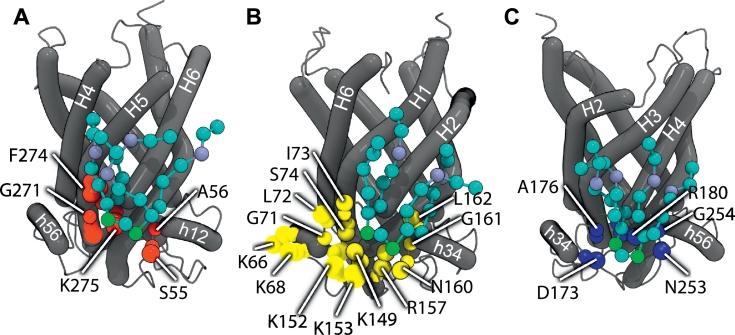
Representative cardiolipin interactions at the three binding sites of yeast AAC3 (grey cartoon) during the coarse-gained simulations: cardiolipin (cyan balls-and-sticks) with phosphate beads (green) and unsaturated bond on the 18:1 cardiolipin acyl chain (purple); and residues interacting with cardiolipin in (A) binding site CDL801 (red), (B) binding site CDL800 (yellow), and (C) binding site CDL802 (blue).

The cardiolipins interacted at distinct sites on the AAC surface. To quantify these binding sites, pairs of residues of the AAC were clustered if they interacted frequently and simultaneously with cardiolipin phosphate groups (Figs. S5 and S6). The AAC residues bound to cardiolipin formed several distinct clusters in both inner and outer leaflets (Fig. 3). In the inner leaflet, three binding sites were identified between matrix helices: (i) h12 and h34 (site CDL800); (ii) h34 and h56 (site CDL802); and (iii) h12 and h56 (site CDL801), and three in sites on each of the matrix helices (sites matrix h12, matrix h34, and matrix h56) (Fig. 3, Fig. 4). In the outer leaflet there were three sites comprising residues from (i) H1 and H6 (site outer 1); (ii) H2, H3, and H4 (site outer 2); and (iii) H5 (site outer 3) (Fig. 3).

Examining the cardiolipin binding sites in the outer leaflet, the residues interacting with the cardiolipin headgroup were located at the termini of the transmembrane α-helices and the loop regions (Fig. 3A). These residues are not conserved in either the AAC subfamily or across the mitochondrial carrier family, suggesting these interactions constitute non-specific interactions of the cardiolipin headgroup with hydrophilic residues in the interfacial region.

At the alternative binding sites of the inner leaflet—sites matrix h12, matrix h34, and matrix h56—the principle interacting residues included basic residues in the matrix helices that are equivalent to each other in the three repeats: Lys-75 and Lys-178 in the yeast AAC2; and Lys-167 and Lys-262 in the yeast AAC3 (Fig. 3). Furthermore, these residues of the matrix helix are well conserved across the mitochondrial carrier family [17].

At the binding sites CDL800, CDL801 and CDL802 of the inner leaflet, cardiolipin headgroups interacted with several residues including the backbone atoms of the glycine and the side chain of the central basic residues of the conserved [YWF][KR]G motif, and the backbone atoms of glycine of the [YF]xG motif (Fig. 3, Fig. 4).

**Fig. 5:**
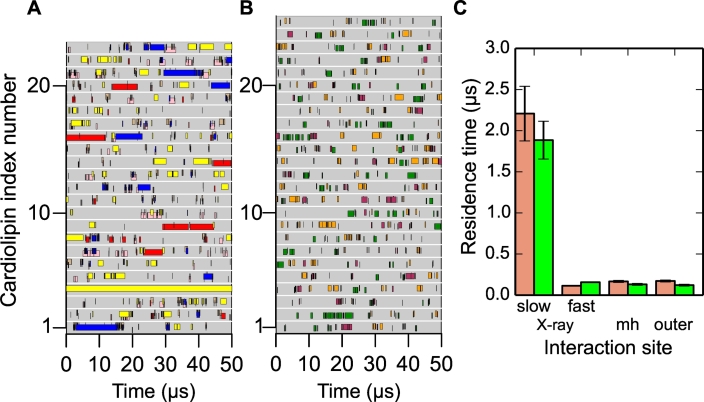
Dynamics of cardiolipin binding to the yeast AAC3 during coarse-grained simulations. Binding of individual cardiolipin phosphates to binding sites of AAC3 in the (A) inner and (B) outer leaflets, and (C) summary of dynamics using residence times for each class of interaction site for all yeast AAC simulations. Each grey block represents a cardiolipin of either the (A) inner leaflet or (B) outer leaflet. Coloured bars on a grey block indicate binding of the cardiolipin phosphate with a specific binding site of the yeast AAC isoform: CDL800 (yellow); CDL801 (red); CDL802 (blue); h12 (cyan); h34 (pink); h56 (brown); outer 1 (green); outer 2 (pale blue); and outer 3 (orange). (C) Residence times for cardiolipin at the three different classes of interaction sites for both simulation repeats of AAC2 (pink) and AAC3 (bright green): at the X-ray crystallography binding sites CDL800, CDL801 and CDL802 (‘X-ray’), the matrix helix binding sites (‘mh’) and the outer leaflet interaction sites (‘outer’). Fitting curves for these values are shown in Fig. S9. X-ray crystallography binding site residence times were fitted best by assuming that cardiolipin interactions were comprised of two populations: fast interactions and slow interactions (see Materials and methods), and calculating their residence times separately.

Having identified interaction sites on the AAC surface, the dynamics of the cardiolipins at these sites were investigated by extracting cardiolipin–protein interaction profiles (Figs. 5A and B, and Figs. S7 and S8) and deriving cardiolipin–protein residence times for each type of interaction site (crystal structure interaction sites; matrix helix interaction sites and outer leaflet interaction sites; Figs. 5C and S9). Cardiolipin binding was longest-lived at the crystal structure interaction sites CDL800, CDL801 and CDL802 (individual interaction times were in the range of tens of microseconds), and these sites were occupied almost continuously, except for binding site CDL802 in the yeast AAC2, which was occupied for 50% of the simulated time (Figs. 5A and B and Fig. S8A). In the sites corresponding to the crystal structure binding sites, residence times showed that cardiolipins had two types of interaction: a population of ‘short’ cardiolipin interactions (in the order of 100 ns) and a population of ‘long’ interactions, on the microsecond time scale (Fig. 5C). The matrix helix cardiolipin interaction sites had residence times in the range 100–200 ns, similar to POPC and POPE (Fig. 2), suggesting they are not cardiolipin binding sites, but may be transient interaction sites as the cardiolipin enters and exits the preferred binding sites (CDL800, CDL801 and CDL802) and help to maintain a ‘reservoir’ of cardiolipin around the AAC. Outer leaflet interaction sites displayed residence times similar to those of POPC and POPE, suggesting that these sites are also transient cardiolipin interaction sites.

To understand in finer detail the cardiolipin–protein interactions identified in the coarse-grained simulations, atomistic simulations were done starting from structures from each of the two coarse-grained simulations of the yeast AAC2 and AAC3. Structures were taken at 34.6 μs and 25.1 μs for the AAC2 (repeats 1 and 2, respectively) and 35.0 μs and 30.1 μs for the AAC3 (repeats 1 and 2, respectively). These points were chosen because cardiolipin occupied all three major binding sites seen in the crystal structures: CDL800, CDL801 and CDL802. All snapshots were reverse-transformed to atomic models [37]. Atomistic simulations of each bilayer system were run for 20 ns and residues interacting with cardiolipins in sites CDL800, CDL801 and CDL802 were noted (Fig. 3). The simulations recapitulated the results of the coarse-grained simulations. For example, interactions were maintained between the phosphate groups and the backbone atoms of the [YWF][KR]G and [YF]xG motifs in the CDL800 and CDL802 sites, and the basic residue of the [YWF][KR]G motif in each repeat continued to bind cardiolipin for the majority of cases.

The cardiolipin acyl chains did not bind to the transmembrane α-helices in a specific way, for instance in the inter-helical grooves. Rather, the density of cardiolipin acyl chains showed a more diffuse interaction in agreement with the crystallography of AAC crystals, which showed density for cardiolipin headgroups but not for the most of the acyl chains (Fig. S10). Acyl chain densities do not extend to the outer leaflet side of the protein (Fig. S10) because the density is too low, most likely due to the very transient nature of the cardiolipin interactions on the outer leaflet side of the protein. Interestingly, the membrane was perturbed around AAC, particularly around the cardiolipin interaction sites, where the membrane is thinner (Fig. S11). The membrane perturbation at these sites also meant that matrix helices and loops on the matrix-facing side were partially solvent exposed, whereas the helices facing the intermembrane space are not (Fig. S11). The results of these simulations agree well with the results of atomic force microscopy imaging [38,39].

### 2.3 Cardiolipin binding to the bovine AAC1

The simulation results of the bovine AAC1 in a cardiolipin-containing bilayer were consistent with those of the yeast AAC simulations (Fig. S12). Residues of the bovine AAC1 that interacted with cardiolipin phosphates were clustered into ‘hotspots’ (Figs. S5 and S6), and were equivalent to the three sets of binding sites identified in the yeast AAC simulations. First, in the outer leaflet cardiolipins bound to residues from transmembrane α-helices H3 and H4 (site outer 2) and residues from helices H1 and H6 (site outer 1) (Figs. 3 and S6). But these residues are not conserved in the AAC subfamily or the mitochondrial carrier family (sites outer 1, 2 and 3)—as with the equivalent yeast binding sites—suggesting non-specific interaction of the cardiolipin headgroups with hydrophilic residues in the interfacial region. Second, in the inner leaflet, cardiolipin bound at sites that overlap with the cardiolipin binding sites in the crystal structure (sites CDL800, CDL801 and CDL802). At these binding sites, several residues bound to the cardiolipin (Fig. 3). But it was notable that the central residue of the [YWF][KR]G motif in each domain (Arg-174, Arg-71 and Gln-174) bound cardiolipin by orienting the side chain so that it was directed at the cardiolipin phosphate, including Gln-174. The interaction with Gln-174 showed that a positively charged residue was not a requirement for cardiolipin binding. Third, cardiolipin bound to residues in the inner leaflet on each of the three matrix helices (sites matrix h12, matrix h34, and matrix h56), including conserved basic residues (Lys-62, Lys-162 and Arg-258) equivalent to those identified in the yeast simulations. In the bovine AAC1 simulations a binding site at matrix h56 was identified, unlike in the yeast AAC simulations (Fig. S5).

Cardiolipin residence times were higher than those of POPC and POPE (Figs. S12C, S13 and S4). Cardiolipin residence times in the inner leaflet with the bovine AAC were shorter than in the yeast AAC simulations, but this is likely due to the shorter bovine AAC simulations not permitting longer binding times of cardiolipin to be sampled as fully as they are for the yeast AACs. Analysis of the dynamics at each interaction site separately showed that the longest-lived cardiolipin interactions with the bovine AAC1 were at the crystal structure binding sites CDL800, CDL801 and CDL802, as with the yeast AACs (Fig. S12D and E and Fig. 5). Two cardiolipin interactions with the bovine AAC1 were maintained for the duration of one simulation (cardiolipin 1 at binding site CDL800 and cardiolipin 4 at binding site CDL802), and several other interactions lasted until the end of the simulation (cardiolipin 9 of system 1, and cardiolipin 23 of system 2, both at binding site CDL801) (Fig. S12D). Residence times for cardiolipin at the matrix helix and outer leaflet interactions sites were little different from POPC and POPE lipids, suggesting they are transient interaction sites, as for yeast AAC.

To investigate in more detail the binding sites found in the coarse-grained simulations, short atomistic simulations were performed of the bovine AAC1 structures obtained from coarse-grained simulations, with snapshots taken at 4 μs for both simulations. Cardiolipins in the binding sites CDL800, CDL801 and CD802 interacted with the same residues identified by using coarse-grained simulations of the bovine AAC1 (Fig. 3).

### 2.4 Trimethylated bovine AAC

The bovine AAC1 is trimethylated at Lys-51 [26], although this is unresolved in the crystal structure. Further simulations were performed with this residue modelled as trimethylated, but the results indicated that the TM-Lys-51 had little effect on cardiolipin interaction in the simulations (Fig. S12). Lipid residence times for the native trimethylated and non-methylated bovine AAC1 were similar (Figs. S12C and S4), as were the binding sites derived from the residue clustering (Figs. S5 and S6); Lys-51 participated in the CDL801 binding site in the non-methylated and trimethylated AAC1 simulations. Although the simulations of the bovine trimethylated AAC1 were shorter than other simulations (4 μs as compared to 10 μs for the non-methylated bovine AAC1, and 50 μs for yeast AAC simulations), the cardiolipin interaction profiles for the bovine trimethylated AAC1 (Fig. S12D) were similar to those in other simulations; in the inner leaflet, the binding sites CDL800, CDL801 and CDL802 were occupied for the duration of the simulation, and in most cases, this was by a single cardiolipin. In particular, the binding site CDL801 containing TM-Lys-51 retained cardiolipin as well as in simulations where Lys-51 was not methylated.

The similarity between the binding sites identified via the clustering method for the bovine non-methylated and trimethylated AAC1 (Figs. S5 and S6) indicated that the method we used to identify binding sites was robust.

## 3 Discussion

Cardiolipin binding sites will have a range of affinities for cardiolipin. Cardiolipins present in crystal structures likely represent binding sites with the strongest interactions since they are not removed during protein purification despite extensive washes. However, cardiolipin may bind with lower affinity to additional sites that are unoccupied in crystal structures, because the cardiolipin is removed during protein purification. For example, cardiolipin is necessary for the optimal activity of the F-ATP synthase [40,41], but is absent from crystal structures [27]. However, we previously showed coarse-grained simulations identified binding sites in the c-rings where cardiolipin associated by using residues that were conserved among the animal kingdom [28].

In this study, molecular dynamics was used to detect cardiolipin binding sites of the AAC. The results showed that the dynamics of cardiolipin binding was different from those of the phospholipids POPC and POPE, which interacted only transiently with the AAC. The major cardiolipin binding sites in the simulations overlapped with those observed in the crystal structures of the AAC in detergent. The shorter-lived cardiolipin binding sites are not observed in the crystal structures and thus are likely to represent transient interaction sites.

### 3.1 Longevity of cardiolipin interactions with the AAC

In simulations presented here, the calculated residence times showed that cardiolipin had significantly longer-lived interactions with the AAC than POPC and POPE phospholipids, agreeing with many experimental findings [[14], [15], [16], [17], [18],^42^,^43^]. When calculating residence times for cardiolipin, some cardiolipin interactions with the AACs were maintained for the duration of the simulation, and so the maximum calculated cardiolipin residence times were limited to the length of the simulation, and thus may be underestimated. This is especially pertinent for the cardiolipins present in the crystal structures of the AAC, which are retained despite extensive washes during purification, and suggest they are bound very tightly.

### 3.2 Identification of cardiolipin binding sites by coarse-grained molecular dynamics

The primary advantage of coarse-grained molecular dynamics over conventional atomistic simulations is the longer simulation time-scales. This advantage enables dynamics of larger molecular systems to be investigated, such as the self-assembly of lipid bilayers and the diffusion of lipids and proteins in bilayers, which is necessary to simulate protein-lipid interactions. The limited time scales of conventional atomistic simulations, in the order of hundreds of nanoseconds, mean that lipids do not have the opportunity to sample new binding sites and often need to be placed around proteins in or very close to binding sites. However, the coarse-grained simulations have disadvantages, as simplification of the system representation is required. Most critically, groups of atoms are represented as beads with particular physiochemical properties. For example, a lysine amino acid side is represented as 3 beads: a single bead for the main chain and two beads for the side chain (a terminal positively charged one, which is bonded to the main chain by an apolar bead); four water molecules are represented by a single coarse-grained water bead; and protein secondary structure is maintained by constraints between beads. Despite these necessary simplifications, coarse-grained simulations have successfully been applied to simulate proteins in biological membranes, to investigate lipid dynamics and protein-lipid interactions [35], and to generate the starting configurations for atomistic simulations [29,37].

In the simulations, cardiolipins started with a random distribution in the lipid bilayer in each coarse-grained model, but rapidly became associated with the AAC during all simulations. The sites CDL800, CDL801, and CDL802 with which cardiolipin interaction was the longest in the simulations coincided with those identified previously in yeast and bovine crystal structures [[16], [17], [18]], validating the use of coarse-grained simulations to study protein–lipid interaction sites [35], and adding to a growing number of studies for cardiolipin [28,[31], [32], [33], [34]]. Notably, our results agree well with recent independent coarse-grained simulations of the yeast and the bovine AAC with cardiolipin, which identified the same three binding sites [34]. A comparison of the residues in the three binding sites identified in our study and that of Hedger et al. is given in Table S1. There are some differences between residues identified, which can be rationalized by differences in the way the binding sites are defined. First, the cardiolipin headgroup is taken as the GL0 bead (representing the glycerol moiety between the two cardiolipin phosphates), whereas in our study we used the two phosphate moieties as the cardiolipin headgroup. Second, Hedger et al. identify interacting residues by their frequency of interaction with the cardiolipin headgroup, whereas in this study the clustering method was used (see Material and methods). The similarity of the binding site residues in the two independent studies further underlines the robustness of the method.

The crystal structure binding sites are formed by the residues shown in Figs. 1 and S1, and cardiolipin headgroups interact with the backbone amine groups of glycine of the [YWF][KR]G motif at the N-termini of even-numbered helices, and conserved [YF]xG motif at the N-termini of the matrix helices [17]. Both the [YWF][KR]G and [YF ]xG motifs are highly conserved across members of the mitochondrial carrier family [17], and it is suggested that all members of the mitochondrial carrier family bind cardiolipin at the same position [17]. However, the side chain of the basic residue of the [YWF][KR]G motif that bound cardiolipin during the simulations (Fig. 3) was interacting with cardiolipin in only a single binding site (AAC2, CDL802) in the crystal structures of the yeast AAC [17] (Figs. 1 and S1). Generally, these basic side chains point away from cardiolipin phosphate groups in the crystal structures and are too far away for strong polar interactions, including hydrogen or ionic bonds (Fig. 1D). In contrast during the simulations, the basic residues were observed to interact with the phosphate groups of cardiolipin. Possible reasons for this discrepancy will be discussed below. However, cardiolipin was also bound at the equivalent site during simulations of the bovine AAC, in which the basic residue is replaced by a glutamine, and demonstrating that the basic residue was not the driver for cardiolipin binding in the simulations. Binding sites defined in the coarse-grained simulation extended further towards the C-termini of the matrix helices, sitting more squarely between the matrix helices than in the crystal structure (Fig. 1, Fig. 3). Cardiolipin binding at sites CDL800, CDL801 and CDL802 in the AACs may be driven by helix dipoles of matrix and even-numbered helices [17]. However, the directional hydrogen bonding network in helices that causes this helix dipole is not represented in the MARTINI force field, suggesting electrostatic and van der Waals interactions also contribute to long-lived cardiolipin interaction. However, the absence of modelled helix dipoles removes constraints for binding, which could cause the cardiolipin binding sites to be less defined in the simulations compared to those in the crystal structures. Another potential disadvantage of course-grained simulations that may affect cardiolipin binding is that each water bead represents four water molecules, so the ability is compromised to simulate directional hydrogen bonds, bridging waters and entry of waters to small cavities and crevices, as observed around the cardiolipin binding sites in the crystal structures of the bovine AAC. It is possible that the side chain of the basic residue of the [YWF][KR]G motif fills the void left by the water leading to small differences in the cardiolipin binding poses between the simulations and crystal structures. In the atomistic simulations that used the conformations from the coarse-grained simulations as a starting point, the side chain of the basic residue of the [YWF][KR]G motif continued to interact with cardiolipin. However, atomistic simulations are dependent on the starting conformations because of the relatively short simulation times, so drawing further conclusions will require an ensemble of atomistic simulations, with a wide variety of starting structures. Notably, in recent atomistic simulations of the AAC and cardiolipin performed by Hedger et al. [34], Arg-71 of the bovine AAC1 does not interact with cardiolipin, demonstrating that this conformation can be sampled in atomistic simulations.

Our coarse-grained simulations identified other transient cardiolipin interaction sites with the AAC in the inner and outer leaflets. In previous simulations of AAC and cardiolipin by Hedger et al. [34], these interaction sites had not been identified, in part because cardiolipin was not included in the outer leaflet and also because Hedger et al. used a different method to identify binding site residues; residues that interact most frequently were identified by Hedger et al., whereas in this study, residue clustering was used (see Materials and methods). The residence times at these transient interaction sites were less than previously found for cardiolipin bound to the c-ring of ATP synthase, which had residence times in the range 200–600 ns [28], and comparable to the residence times for the POPC and POPE interaction with the AAC, suggesting they are non-specific interactions. Thus, while we show that cardiolipin has diverse dynamic interactions with AAC, we identify that AAC only has three specific cardiolipin binding sites, in agreement with the X-ray crystallography and NMR/ESR results [[14], [15], [16], [17], [18]].

### 3.3 The possible role of dysfunctional cardiolipin binding in mitochondrial diseases

Of the three characterised diseases caused by dysfunctional AAC, none have mutations in the cardiolipin binding site formed by [YWF][KR]G and [YF]xG motifs [44]. However, the cysteine replacements of the aromatic residue in each of its three [YWF][KR]G motifs led to an inactive bovine oxoglutarate carrier [45], and in case of the human mitochondrial thiamine pyrophosphate carrier, mutation p.Gly177Ala has been associated with Amish microcephaly [46], and in case of the human mitochondrial aspartate/glutamate carrier, mutation p.Gly393Ser causes citrin deficiency [47]. These observations indicate that impaired binding of cardiolipin may lead to mitochondrial diseases due to increased protein instability [19] and/or lower transport rates [48].

The role of trimethylation of Lys-51 in the bovine AAC—and likely the other metazoan AACs—was not apparent from the simulations. Other mitochondrial proteins also show lysine trimethylation, including the c-ring of the ATP synthase in a diverse range of metazoa [49,50]. However, simulations of the trimethylated ATP synthase c-rings also did not identify a conclusive role of lysine trimethylation [28], yet its conservation among metazoa suggest that there must be an important physiological reason that has not been resolved.

Without a crystal structure of the m-state (i.e. outward facing state) it is difficult to speculate how cardiolipin might interact with this state, and thus to provide insight into the effect of cardiolipin on the translocation event.

## 4 Conclusions

The only long-lived cardiolipin binding sites of both the bovine and the yeast AACs were consistent with those identified previously in crystal structures [[16], [17], [18]], and had interaction times in the order of tens of microseconds. These results show that multiscale simulations can be used for the identification of cardiolipin interaction sites. Although the role of cardiolipin in the function of the AACs remains unresolved, their conserved binding sites indicate that they are important for the mechanism, which is conserved across the mitochondrial carrier family. Given that cardiolipin is required for optimal functioning of the AAC, these studies may provide a starting point for investigating the molecular mechanism and pathophysiology of mitochondrial carriers.

## 5 Materials and methods

### 5.1 Simulation programs, force fields and parameterisation

Coarse-grained simulations were performed using GROMACS (versions 4.5.3 and 4.5.4) [51] and the MARTINI force field [[52], [53], [54]], with the MARTINI elastic network model parameterization (ElNeDyn) [55] used for protein structures. Because the MARTINI coarse-grained parameterisation both reduces the time taken to run simulations, and speeds up dynamics compared to atomistic simulations, the recommended time conversion factor of four [53] was used to convert MARTINI simulation times to more meaningful effective times for the calculation of dynamic properties.

Coarse-grained simulations were performed using periodic boundary conditions with constant number of particles, constant pressure and constant temperature (NPT ensemble). Temperature was coupled separately for lipids, protein and water to a thermostat at 296 K by using the Berendsen algorithm and coupling constant of 0.3 ps. Pressure was coupled semi-isotropically to a Berendsen barostat at 1 bar with compressibility of 3 × 10^−5^ bar^−1^, coupling constant of 8 ps, and a time step of 20 fs. As is standard with the MARTINI force field, non-bonded interactions were treated with a switch function over 0.0–1.2 nm (electrostatic interactions) and 0.9–1.2 nm (van der Waals), and a dielectric constant of 15 [52,53].

As cardiolipin is not part of the standard MARTINI parameter set, the tetraoleoyl cardiolipin parameters of Dahlberg and Maliniak were used [56,57]. The cardiolipin in the simulations carried a double negative charge, as reported at physiological pH [4,5], and matching a survey of cardiolipin–protein interactions for structures in the Protein Data Bank (PDB) [58] showing cardiolipin headgroup geometries are most consistent with a double-negatively charged cardiolipin [59]. The cardiolipin model has four 18:1 acyl groups. Parameters for a coarse-grained model of trimethyllysine (TM-Lys) were based upon a model using MARTINI parameters for lysine and choline [28]. For atomistic simulations the all-atom CHARMM-36 force field was used [60], also run using GROMACS, using the cardiolipin model developed by Lemmin et al. [61]. Simulations were visualised by using VMD [62] and figures from simulations were prepared by using PyMOL [63] and Bendix [36].

### 5.2 Coarse-grained simulations

#### 5.2.1 Construction of protein models

The AAC structures used in the simulation were from the two *S. cerevisiae* AAC isoform structures (PDB accession number: **4C9G**; and PDB accession number: **4C9Q**) [17] and the bovine AAC1 (PDB accession number: **1OKC**) [16]. Coarse-grained structures of the AACs were built from the PDB structures by using the martinize script (version 2) from the MARTINI website (http://md.chem.rug.nl/cgmartini/index.php/downloads/). Loop-region residues absent in the structures of the *S. cerevisiae* AAC were added by using PyMOL [63] and energy minimised. In the crystal structure of the bovine AAC, the C-terminal residues 294 to 297 of the sixth transmembrane helix (TMH) are absent. These were added by using MODELLER [64], and modelling their secondary structure on the previous four residues of the sixth transmembrane helix. Side chains absent in the crystal structures of the bovine and the yeast AAC isoforms were added by using the mutagenesis tool of PyMOL.

#### 5.2.2 Construction of lipid bilayer models

The lipid bilayer was composed of POPC, POPE and tetraoleoyl cardiolipin in a ratio of about 9:7:4, mimicking the average lipid composition of inner mitochondrial membranes from guinea pig liver, rat liver and pig heart [65]. An initial bilayer was constructed from 125 lipid molecules (56 POPC, 44 POPE, and 25 cardiolipins) by first placing lipids randomly in a simulation box, with six times as many coarse-grained water beads. Lipid bilayers then self-assembled during a simulation of 60 ns, with pressure coupled isotropically using a Berendsen barostat (coupling constant of 3 ps), and temperature with a Berendsen thermostat (coupling constant of 0.3 ps). The self-assembled bilayer was then simulated for a further 15 ns. A larger bilayer of 250 lipids was assembled by pasting together two of the smaller self-assembled bilayers. A second bilayer model was generated by simulating the first bilayer for a further 300 ns.

#### 5.2.3 Insertion of the protein models into the lipid bilayer models

To generate two systems of each AAC for simulation, each coarse-grained model of an AAC was energy minimised in a vacuum for 500 steps, inserted into each bilayer model by using INFLATEGRO [66] (with minor modifications for use with coarse-grained particles), and then solvated.

#### 5.2.4 Energy minimisation and equilibration simulations

Each system was energy minimised in two stages. First, only water was unrestrained, whilst lipid and the AAC were restrained with a force constant of 10,000 kJ mol^−1^ nm^−2^. Second, both water and lipid molecules were unrestrained, but the AAC remained restrained (with the same force constant as before). The systems were then equilibrated as described in the MARTINI ElNeDyn tutorial. For the first stage of temperature equilibration, a short simulation of 2 ns was run, with a time step of 1 fs, followed by a simulation of 4 ns with a time step of 20 fs. Temperature equilibration was run using the NPT ensemble (constant number of particles, constant volume, and constant temperature). A stage of pressure equilibration was run for 1200 ns, again with a time step of 20 fs. Throughout the equilibration phases, the protein was restrained with a force constant of 10,000 kJ mol^−1^ nm^−2^. In a last phase of equilibration, the system was simulated for 120 ns with only the AAC backbone beads restrained.

### 5.3 Production simulations

Production simulations for data collection were run for 50 μs for the yeast AAC2 and AAC3, 10 μs for the non-methylated bovine AAC1, and 4 μs for the bovine trimethylated-Lys-51 AAC1.

### 5.4 Analyses of coarse-grained simulations

#### 5.4.1 Interaction and residence times of lipids

To prevent interaction times being confounded by small and quick on-off motions of protein–lipid interactions (caused by thermal noise), the AAC–lipid distances were smoothed by a 50-point running average, equivalent to a running average over 60 ns.

The AAC–lipid distances were calculated from co-ordinates collected every 1.2 ns from production simulations. A lipid headgroup was considered interacting with the AAC if at least one phosphate bead was interacting with any AAC bead. A whole lipid was considered to be interacting with AAC if at least four lipid beads were interacting with any AAC beads. The lipid (headgroup) interaction time was the length of time that a lipid (headgroup) was continuously interacting with the AAC. Thus, over the course of the simulation, there can be more than one interaction time per lipid.

To characterize the dynamic behaviour of lipid types with regard to interaction times, residence times were determined. The residence time, *θ*, of a lipid type (cardiolipin or POPC/POPE) is defined as the average time a single lipid spends continuously interacting with an AAC. Residence times were calculated from the normalised survival time-correlation function, *σ*(*t*) [31,32,67,68]:

$$\sigma \left(t\right)=\frac{1}{N_{j}}\;\frac{1}{T-t}\;\sum_{j=1}^{N_{j}}\sum_{\nu =0}^{T}\varrho_{j}\left(\nu ,\nu +t\right)$$

where *T* is the total simulation time and *N*_*j*_ is the total number of a lipid type with non-zero interaction time. The function *ρ*_*j*_(*v*, *v* + *t*) has value 1 if lipid *j* continuously interacts with the AAC from time *v* to time *v* + *t* (inclusive), and 0 otherwise. The value of *v* ran from 0 ns to *T* ns in steps of 1 ns, and the values of *σ*(*t*) were determined for every value of *t* from 0 to *T* ns, at 1 ns intervals. *σ*(*t*) was normalised by dividing by *σ*(0), so that the survival time-correlation function has value 1 at *t* = 0. The normalised time-correlation function was modelled as a single exponential function with rate parameter 1/*θ*:

$$\sigma \left(t\right)∼\exp\left(-t/\theta \right)$$

or as a sum of exponential functions for the residence times for interaction sites CDL800, CDL801 and CDL802:

$$\sigma \left(t\right)∼\text{Aexp}\left(\left(-t/\theta_{1}\right)+\text{Bexp}\left(-t/\theta_{2}\right)\right)$$

The value of *θ* (or *θ*_*1*_ and *θ*_*2*_) was determined by fitting the values of *σ*(*t*) to an exponential curve (or sum of exponentials) using the non-linear least squares fitting function (nls) in the R statistical package [69]. For the interaction sites CDL800, CDL801 CDL802, where two residence time parameters are obtained—representing populations of short and long interaction times—the residence time of the long interactions is reported unless otherwise stated.

Residence times from molecular dynamic simulations have been used before in the study of protein–water interactions [67,68,[70], [71], [72], [73]], ion coordination [74], and cardiolipin binding sites on the surface of complexes III and IV [31,32]. In these studies, there is often a specific binding site, or number of different sites on the protein surface. In this study, the surfaces of the AAC exposed to the inner and outer leaflet lipid headgroups were considered as two separate sites. To obtain the overall residence time for a lipid type, these two values were averaged.

The error on residence times was calculated by using a bootstrap method. Calculating an error by using block averaging was inappropriate since, for a 50 μs simulation, the size of time blocks taken would be less than some residence times. Residence times were calculated by fitting the time correlation function to an exponential decay curve. To calculate an error for a residence time, the given set of lipids was resampled 1500 times, and for each resampled group a time correlation function derived and the residence time was calculated, generating a distribution of residence times. The error was taken as the corrected standard deviation of this bootstrap-generated distribution.

### 5.5 Structural analyses of proteins

Root-mean-square deviations (RMSD) were calculated by using GROMACS analysis tools.

To cluster protein residues into binding sites, a distance metric, d_l_(res_i_,res_j_), was defined between any two residues, res_i_ and res_j_, in leaflet l, as:

$$d_{l}\left({\mathrm{res}}_{i},{\mathrm{res}}_{j}\right)=1-\frac{1}{N_{\mathrm{int},l}}\sum_{n=1}^{N_{\mathrm{CDL},l}}\sum_{t=0}^{T}\chi_{n,t}\left({\mathrm{res}}_{i},{\mathrm{res}}_{j}\right)$$

where: χ_n,t_(res_i_,res_j_) is 1 if phosphate beads of the n^th^ cardiolipin are within 0.7 nm of both res_i_ and res_j_ at time point t, and 0 otherwise; N_int,l_ is the total number of interactions between any residue of the AAC and cardiolipin from leaflet l (i.e. either the inner or outer leaflet); N_CDL,l_ is the number of cardiolipin in a given leaflet, l; and T is the total length of the simulation.

Distance metric values range between 0 and 1, where a distance of 1 means that no single cardiolipin interacts simultaneously with the given pair of residues. A low distance metric value indicates that cardiolipins frequently interact (via their phosphate beads) simultaneously with the given pair of residues. For each pair of simulations performed of an AAC, the distance matrices were averaged using a weighted average. With the distance matrix of residue pairs defined, residues were clustered using single linkage clustering by using the hclust function from the R statistical package [69].

### 5.6 Atomistic simulations

Starting structures for atomistic simulations were generated from coarse-grained simulation snapshots by using reverse transformation of a coarse-grained protein model, performed with MARTINI tools [37]. Snapshots from the coarse-grained simulations were chosen such that cardiolipins were interacting at the binding sites: CDL800, CDL801 and CDL802. After reverse transformation, the system was energy minimised and equilibrated by molecular dynamics.

The CHARMM-36 [60] force field was used as this has been most rigorously tested for the cardiolipin atomistic model (developed by Lemmin et al. [61]). Water was modelled using the TIP3P parametrisation [75].

Simulations used periodic boundary conditions with a constant number of particles, constant pressure and constant temperature (NPT ensemble), and a time step of 2 fs. Temperature was coupled separately for lipids, the AAC and water to a thermostat at 300 K using the Nosé–Hoover thermostat and coupling constant of 0.5 ps. Pressure was coupled semi-isotropically to the Parrinello–Rahman barostat at 1 bar with compressibility of 4.5 × 10^−5^ bar^−1^ and coupling constant of 5 ps. Van der Waals interactions were treated with a switch function between 0.8 and 1.2 nm. Electrostatic interactions were computed using the Particle-mesh Ewald (PME) summation, with a grid spacing of 0.12 nm. C-α atoms of the AAC backbone were restrained with a force constant of 1000 kJ mol^−1^ nm^−2^.

### 5.7 Analysis of atomistic simulations

To identify amino acid residues of the AAC that formed binding sites for cardiolipin, the frequency with which cardiolipins interacted with each residue was counted. A residue was considered interacting with a cardiolipin headgroup when any of the cardiolipin phosphate atoms were within 3 Å of any atom of the given residue.

## Transparency document

Transparency document.

## Appendix A Supplementary data

Supplementary material Image 1
