# Supplementary material for: Cardiolipin dynamics and binding to conserved residues in the mitochondrial ADP/ATP carrier
Source: Biochim Biophys Acta. 2018 May;1860(5):1035–45. doi: 10.1016/j.bbamem.2018.01.017 (PMC5988563; doi:10.1016/j.bbamem.2018.01.017)
Supplement: Supplementary file 2 — Supplementary material [file mmc1.docx]

**­­­Supplementary data**


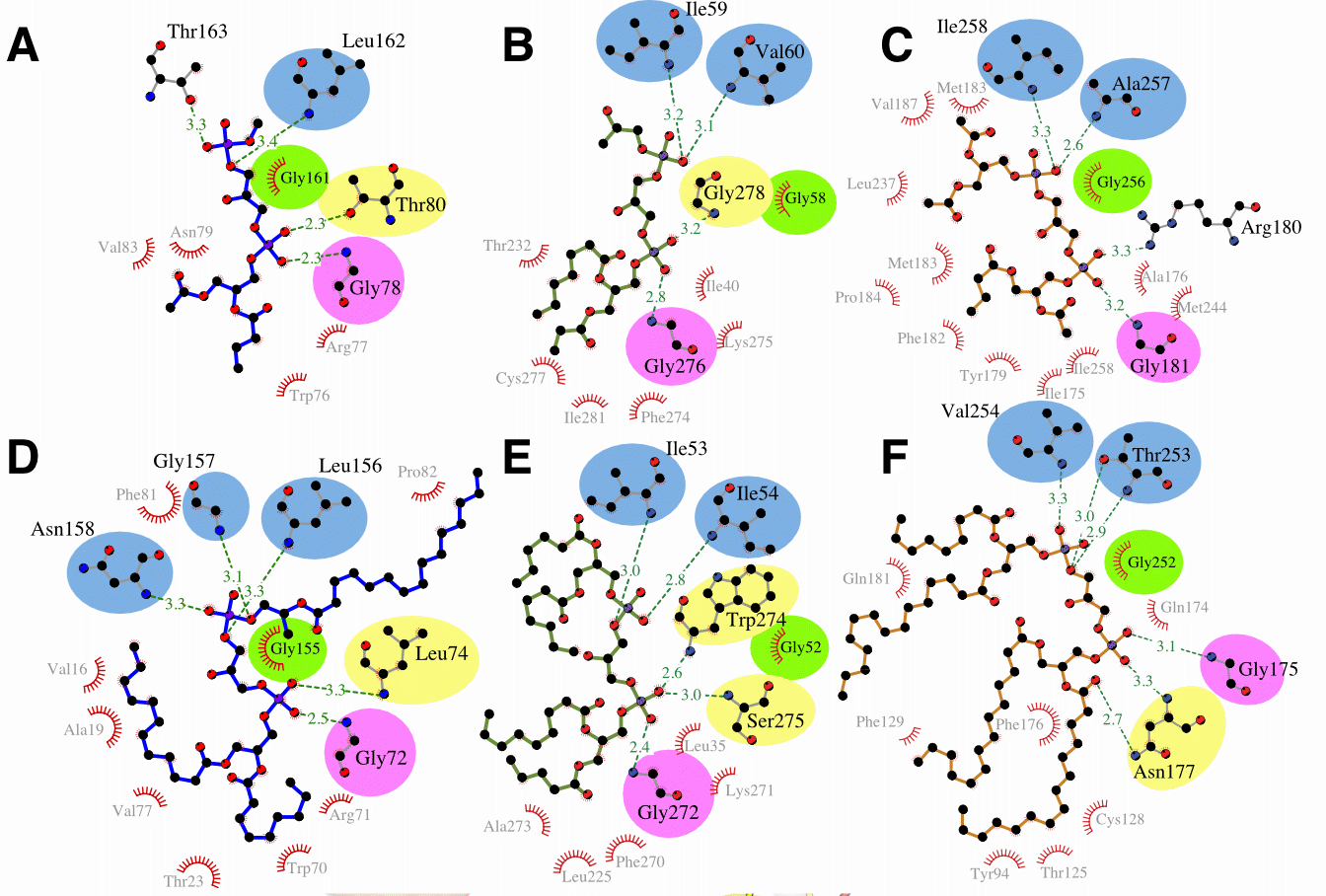


**Fig. S1.** Cardiolipin–protein interactions in structures of yeast and bovine AAC proteins. Schematic diagrams showing interactions of (A-C) yeast AAC3 (PDB ID: 4C9Q chain A) and (D-F) bovine AAC1 (PDB ID: 2C3E) with cardiolipin CDL800 (blue), cardiolipin CDL801 (green) and cardiolipin CDL802 (orange). Highlighted interactions: hydrogen bonds (green dashed lines) with their length (Å); residues in hydrophobic contact with cardiolipin (red arcs with spokes radiating towards the atoms they contact). Coloured ellipses highlight amino acids of: the N-terminal end of the matrix helices (blue); the N-terminal end of the even-numbered transmembrane helices (yellow); the [YWF][KR]G motif (violet); and the [YF]xG motif (green).


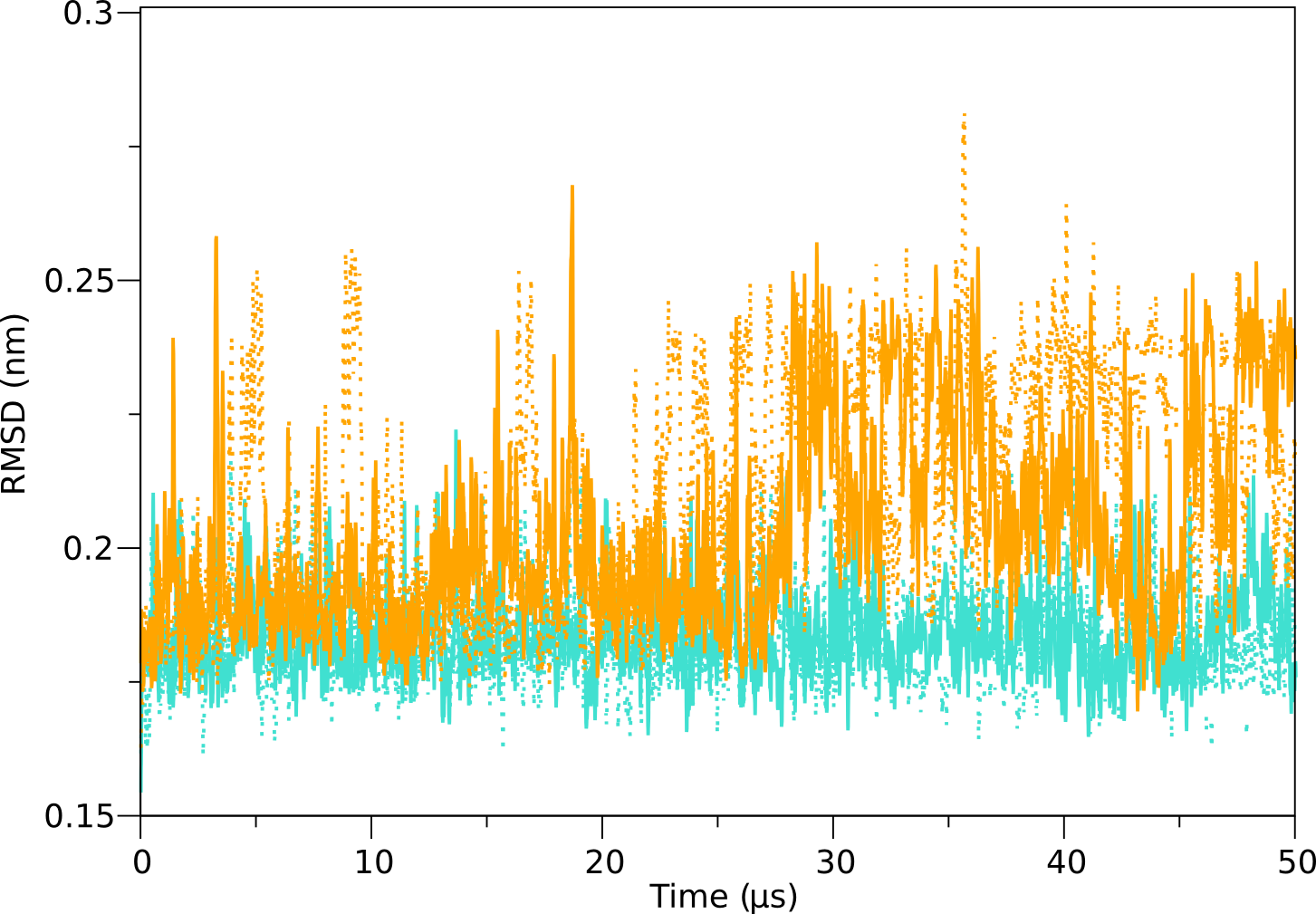


**Fig. S2.** The root mean squared deviation (RMSD) during coarse-grained simulations of the two yeast ADP/ADP carrier isoforms: AAC2 (cyan) and AAC3 (orange). Two systems were built and simulated for each isoform; the first system for each protein is shown as a solid line, the second as a dotted line. The RMSD (shown as the 50 point running average) is measured with respect to each starting structure over the course of the production simulations. Stability of protein structures was indicated by their RMSD remaining within 0.3 nm of the starting structure.


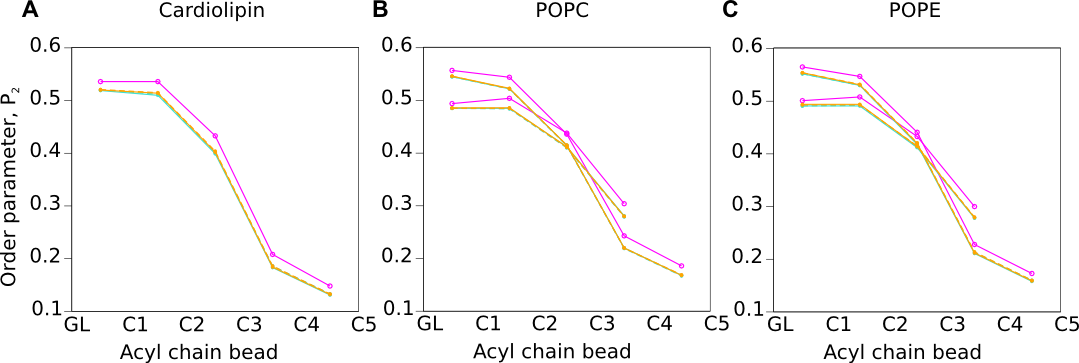


**Fig. S3.** Order parameters for the acyl chains of cardiolipin, POPC and POPE. The parameters for each lipid type were calculated for a pure bilayer (magenta) and bilayers containing either yeast isoform AAC2 (cyan) or AAC3 (orange) for (A) cardiolipin; (B) POPC; and (C) POPE. For cardiolipin, lipid order parameters were averaged between acyl chains, whereas POPC and POPE have two chains of differing lengths and are shown separately. The lipid order parameters demonstrate that bilayers were in the liquid phase, and are in good agreement with previous simulations of cardiolipin-containing bilayers ^27-30^. Lipid order parameters for the pure lipid bilayer were slightly higher than for simulations of the bilayer containing the AACs, indicating presence of the AACs did not cause lipid tails to become more ordered.


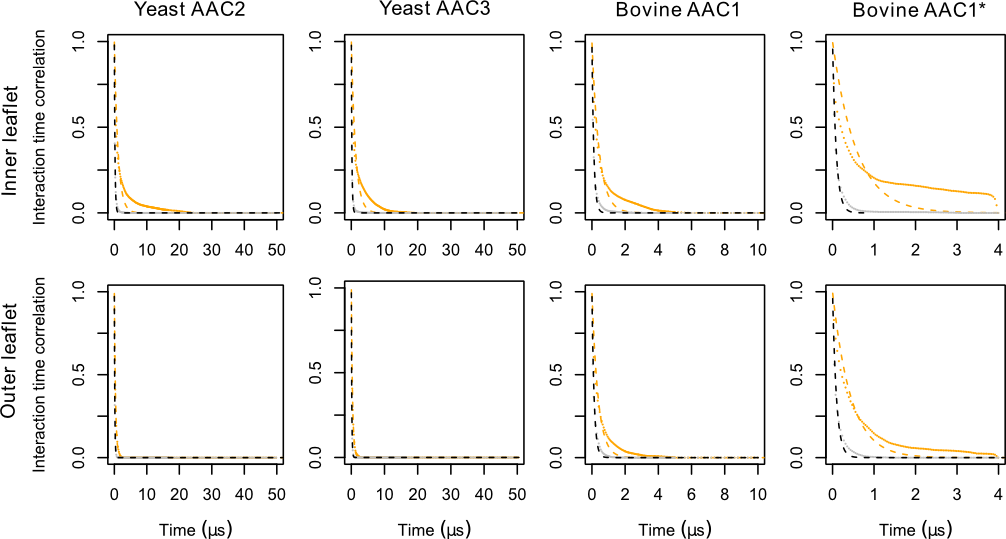


**Fig. S4.** Whole-lipid residence time-correlation functions for the time span of binding between lipids and the whole surface of the AACs. Time-correlation curves derived from simulation data of each AAC simulation, along with their best fit exponential decay curves (dashed lines), are shown separately for lipids of the inner (top) and outer (bottom) leaflets. AAC1* denotes bovine AAC1 with trimethylated TM-Lys-51.


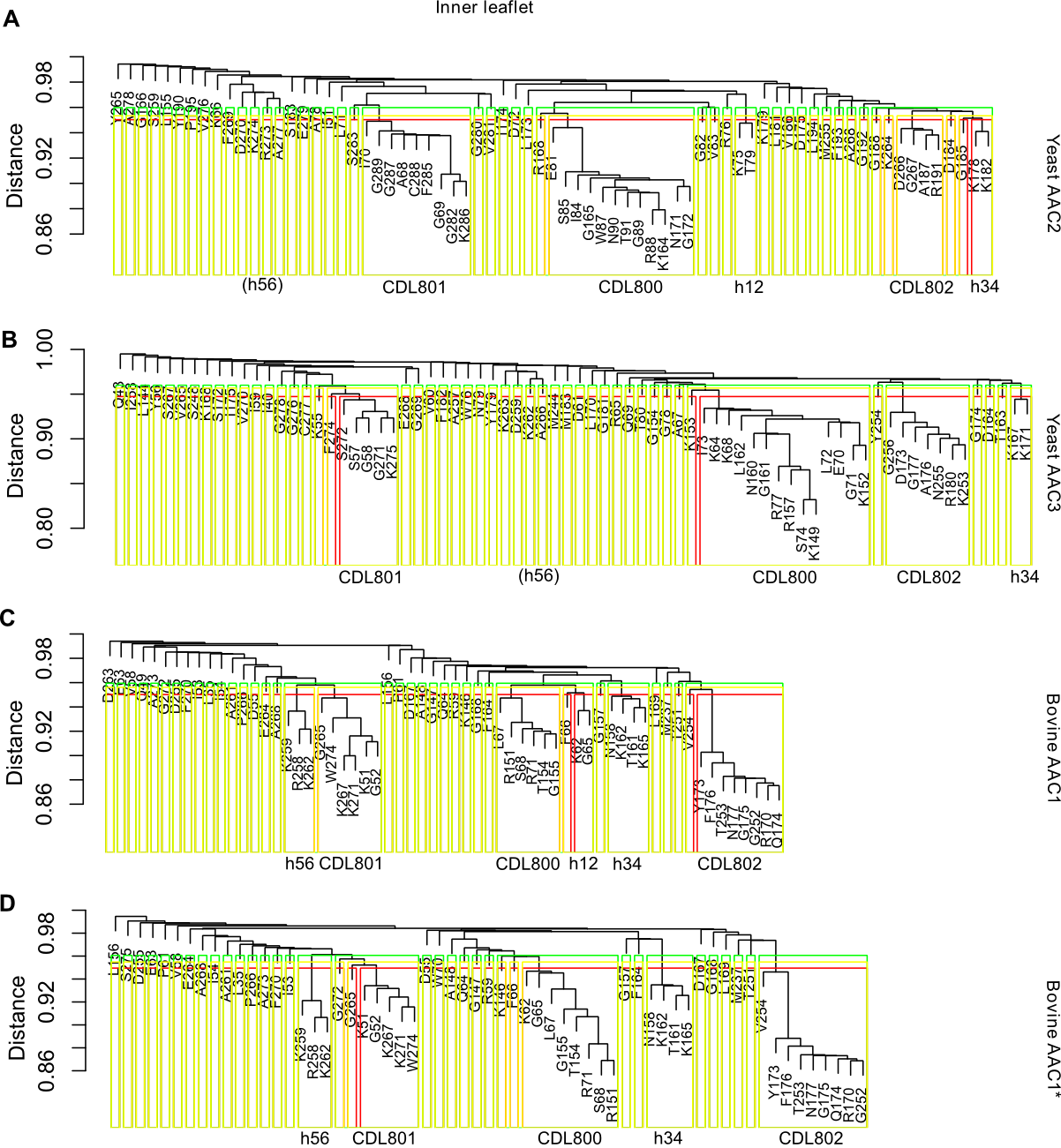


**Fig. S5.** Clustering of surface residues of each AAC according to the frequency of their simultaneous interactions with any single cardiolipin molecule in the inner leaflet. Residues that cluster are more likely to interact simultaneously with cardiolipin and constitute a binding site. Clustering was done for simulations of (A) yeast AAC2; (B) yeast AAC3; (C) bovine AAC1 with demethylated Lys-51; and (D) bovine AAC1 with trimethylated TM-Lys-51. Single-linkage clustering of the residues is according to their pairwise ‘distances’, where the distance between any pair of residues is one minus the proportional frequency of interactions when a single cardiolipin interacted simultaneously with the given pair of residues (see Methods). Dendrograms are cut-off at a distance of ∼0.99, since above this cut-off additions to the tree are only by single nodes. Marked onto the dendrograms are clusters formed at a variety of cut-off distances: 0.96 (green); 0.955 (yellow); and 0.95 (red). AAC1* denotes bovine AAC1 with TM-Lys-51.


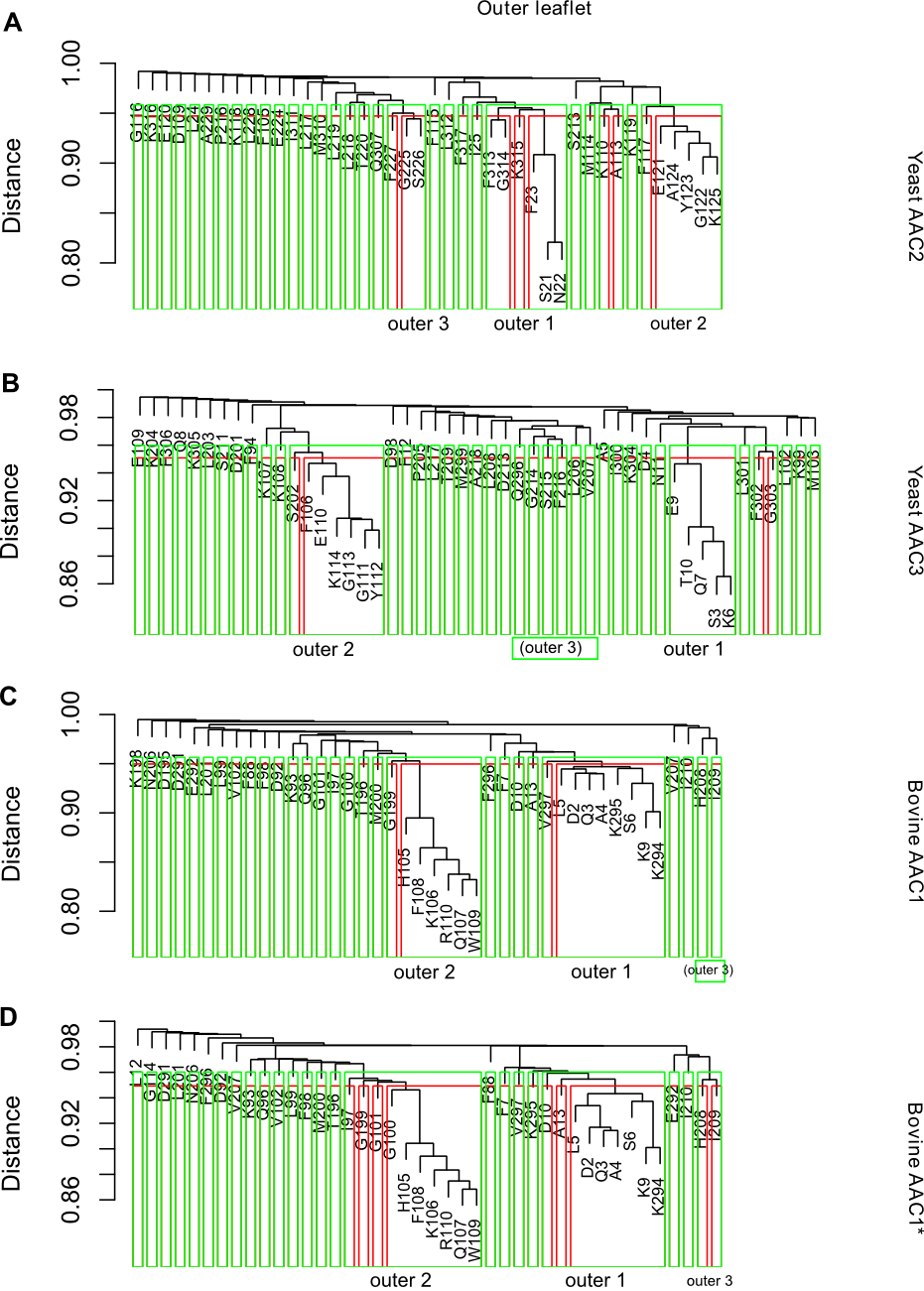


**Fig. S6.** Clustering of surface residues of each AAC according to the frequency of their simultaneous interactions with cardiolipin in the outer leaflet. Clustering was done for simulations of (A) yeast AAC2; (B) yeast AAC3; (C) bovine AAC1 with demethylated Lys-51; and (D) bovine AAC1 with trimethylated TM-Lys-51. Single-linkage clustering of the residues is according to their pairwise ‘distances’ (see legend of Fig. S8). Dendrograms are cut-off at a distance of ∼0.99, since above this cut-off additions to the tree are only by single nodes. Marked onto the dendrograms are clusters formed at cut-off distances of: 0.96 (green) and 0.95 (red). AAC1* denotes bovine AAC1 with trimethylated TM-Lys-51.


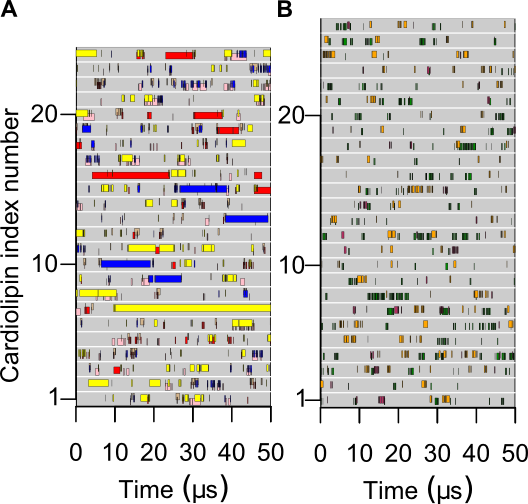


**Fig. S7.** Binding of individual cardiolipin phosphates to binding sites of the yeast AAC3 during coarse-grained simulation. Each grey block represents a cardiolipin of either the (A) inner leaflet or (B) outer leaflet. Coloured bars on a grey block indicate the time span of binding of the cardiolipin phosphate with a specific binding site of the yeast AAC3: CDL800 (yellow); CDL801 (red); CDL802 (blue); h12 (cyan); h34 (pink); h56 (brown); outer 1 (green); outer 2 (pale blue); and outer 3 (orange).


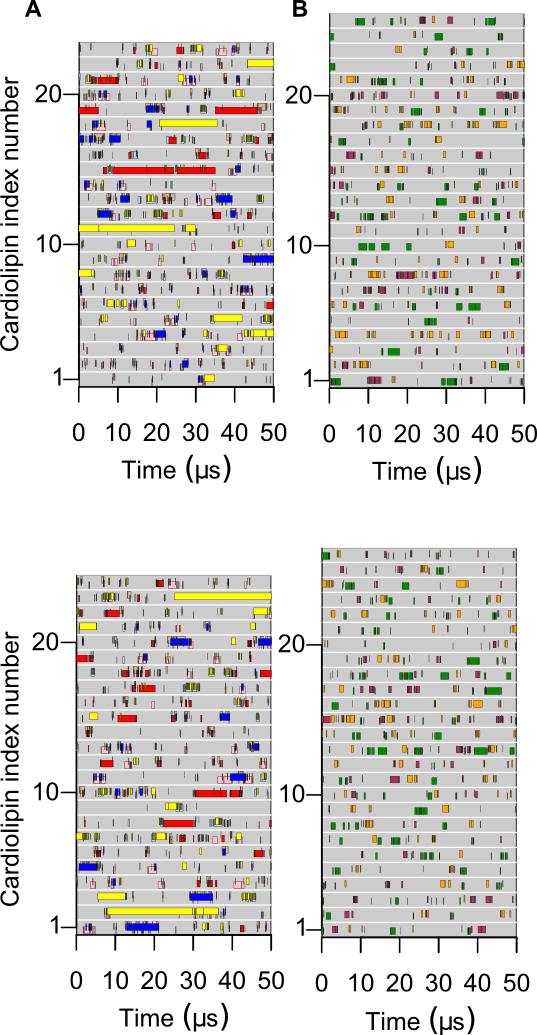


**Fig. S8.** Binding of individual cardiolipin phosphates to binding sites of the yeast AAC2 during coarse-grained simulations. Each grey block represents a cardiolipin of either the (A) inner leaflet or (B) outer leaflet. Coloured bars on a line indicate binding of the cardiolipin phosphate with a specific binding site of the yeast AAC2: CDL800 (yellow); CDL801 (red); CDL802 (blue); h12 (cyan); h34 (pink); h56 (brown); outer 1 (green); outer 2 (pale blue); and outer 3 (orange).


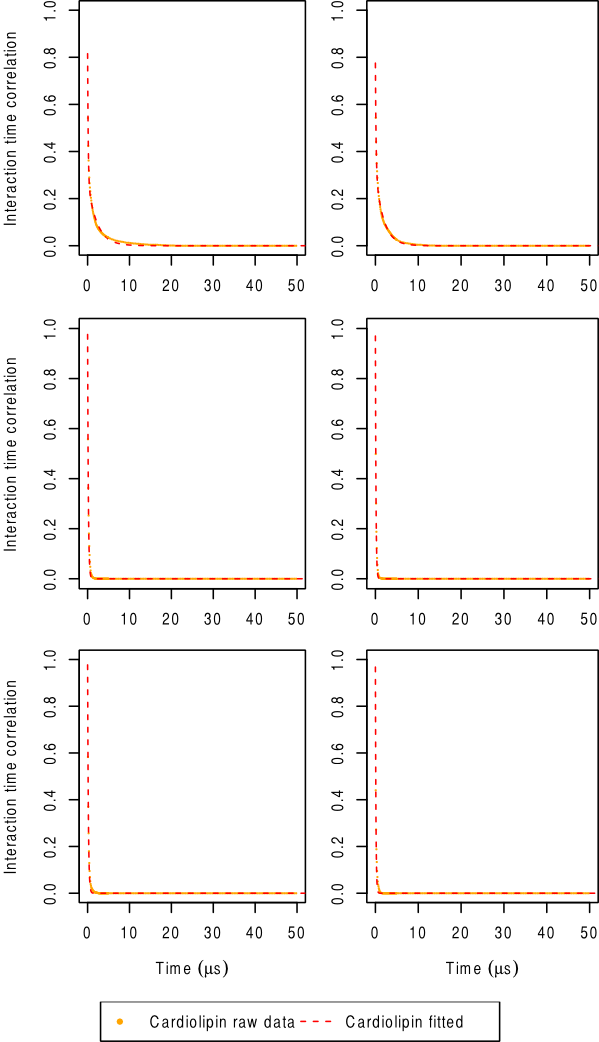


**Fig. S9.** Cardiolipin residence time-correlation functions for the time span of binding between lipids and different binding sites. Time-correlation curves derived from simulation data of each AAC simulation, along with their best fit exponential decay curves (dashed lines), are shown separately for AAC2 (left column) and AAC3 (right column) for different classes of binding sites: crystal structure binding sites (top row); matrix helix binding sites (middle row); and outer leaflet interaction sites (bottom row). Parameters from the fitted graphs define the residence times shown in Fig. 5C and Table 2.

**
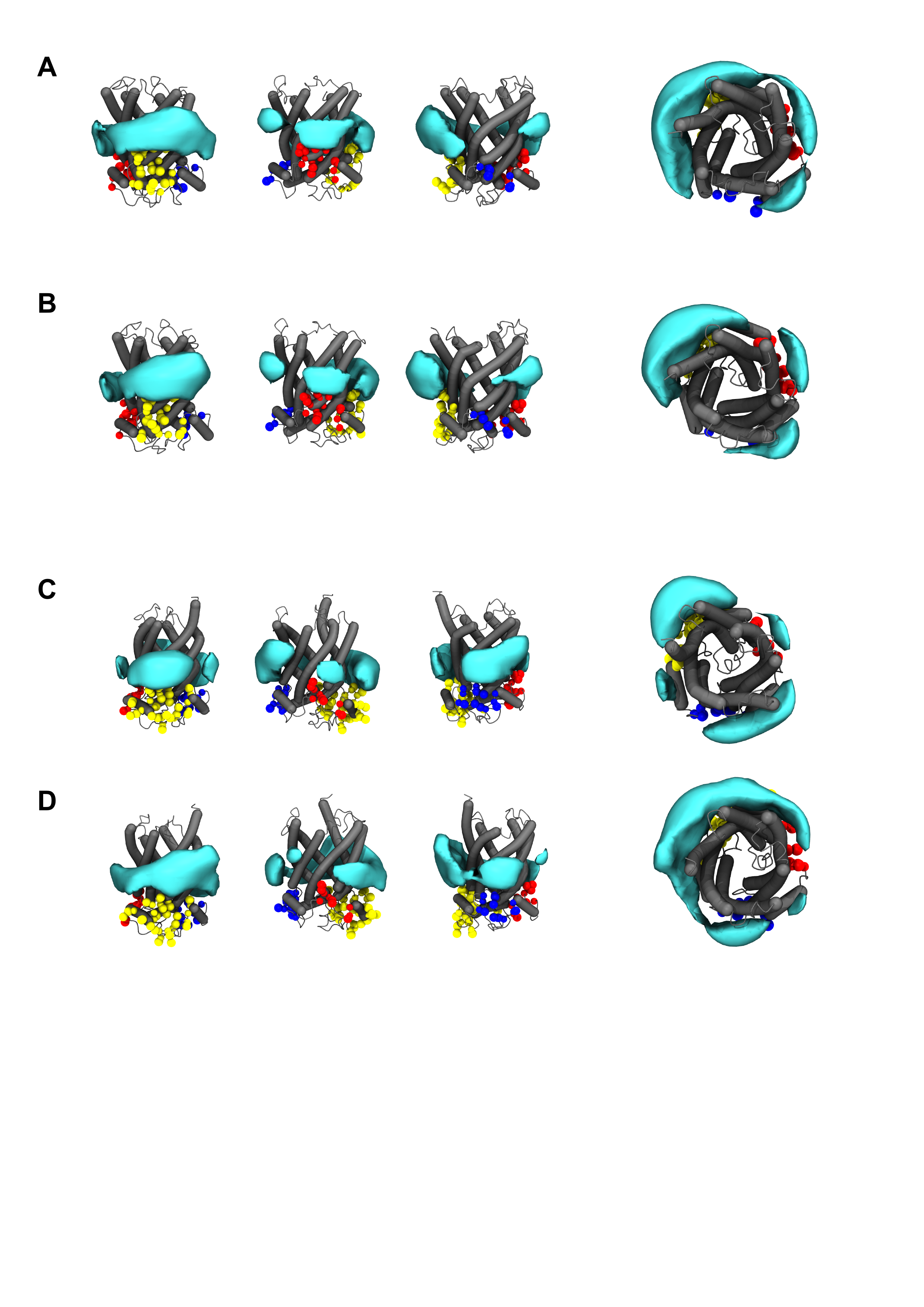
**

**Fig. S10.** Time-averaged densities of cardiolipin acyl chains around AAC2 (repeats 1 and 2; A and B, respectively) and AAC3 (repeats 1 and 2; C and D, respectively) with cardiolipin acyl chain densities shown in cyan. Each panel shows several view of the same simulation: CDL800 to the fore (first column binding site residues as yellow spheres); CDL801 (second column, binding site residues as red spheres); CDL802 (third column, binding site residues as blue spheres) and a view from the cytoplasmic side. Densities are averaged over the course of the each simulation and obtained using the VMD plugin VolMap with the atom radius set to 2 Å and grid size at 3x the particle size.


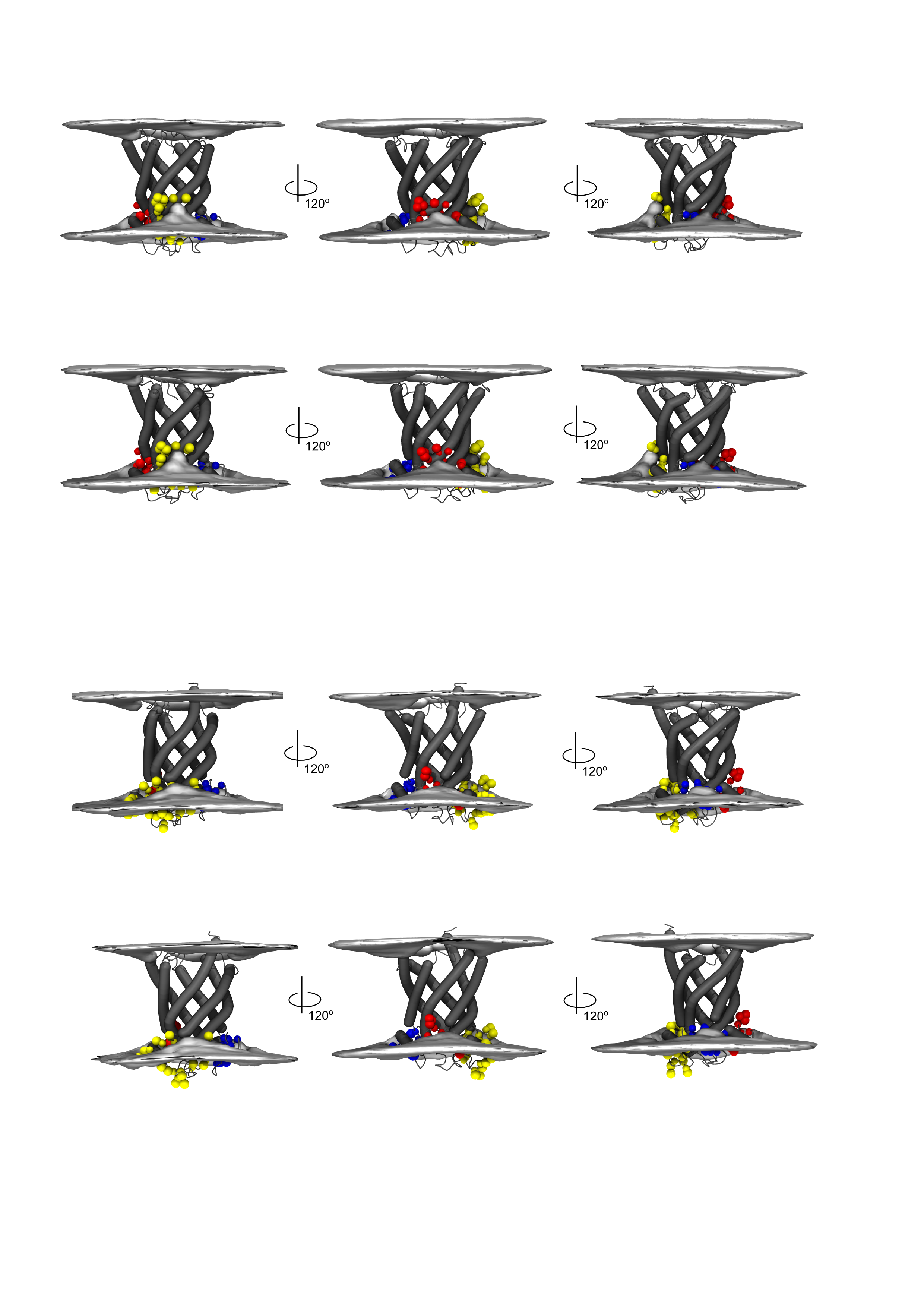


**Fig. S11.** Time-averaged densities of all lipid phosphate groups in relation to AAC2 (repeats 1 and 2; top two rows) and AAC3 (repeats 1 and 2; bottom two rows), with phosphate densities shown in grey. Each row shows several view of the same simulation: CDL800 to the fore (first column, binding site residues as yellow spheres); CDL801 (second column, binding site residues as red spheres); and CDL802 (third column, binding site residues as blue spheres). Densities are averaged over the course of the each simulation and obtained using the VMD plugin VolMap with the atom radius set to 2 Å and grid size at 3x the particle size.


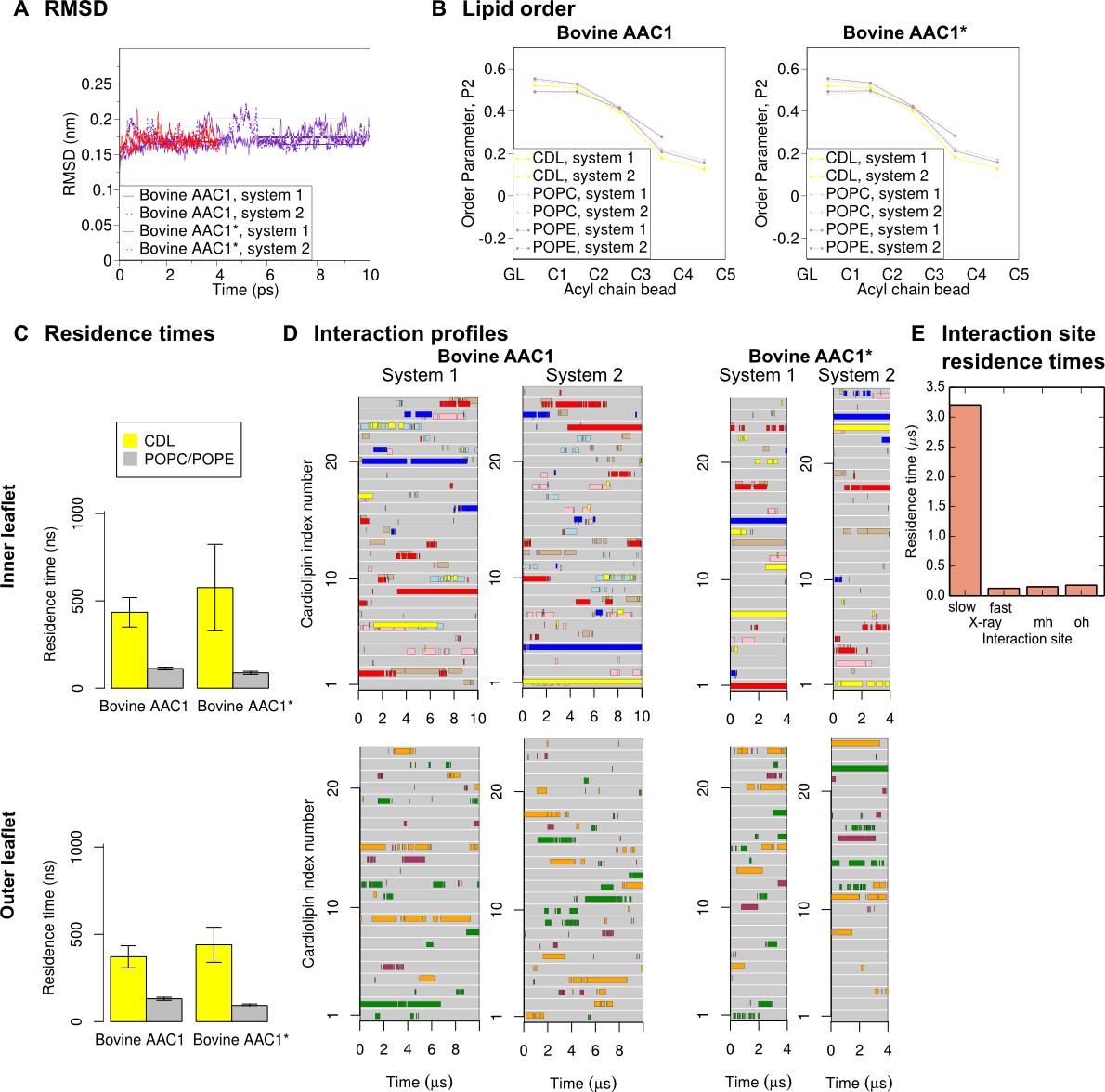


**Fig. S12.** Summary of interactions of cardiolipin with the bovine AAC1 with demethylated Lys-51 and with trimethylated TM-Lys-51 (denoted AAC1* in all panels). (A) The RMSD from the starting structure of the AAC1 with demethylated Lys-51 (purple) and trimethylated TM-Lys-51 (red) over the course of the simulations (fifty point running average). (B) Lipid order parameters in simulations of the demethylated AAC1 crystal structure (left) and the trimethylated TM-Lys-51 AAC1 (right) for acyl chains of cardiolipin (averaged between tails for cardiolipin) (yellow), and with chains shown separately, POPC (grey) and POPE (brown). (C) Residence times for cardiolipin (yellow), and POPC/POPE phospholipids (grey) in the bilayer, shown separately for both inner and outer leaflets. (D) Individual cardiolipin phosphate interactions with distinct binding sites of the AAC1. Each grey block represents a cardiolipin of the inner leaflet (top row) or the outer leaflet (bottom row). Coloured bars indicate binding of the phosphate beads of the given cardiolipin with AAC1 at the binding site specified by the bar colour (see scheme of Figure 5). (E) Residence times for cardiolipin at the three different classes of interaction sites for both simulation repeats of AAC1: at the X-ray crystallography binding sites CDL800, CDL801 and CDL802 (labeled ‘X-ray’); the matrix helix binding sites (labeled ‘mh’); and the outer leaflet interaction sites (labeled ‘outer’). Residence times of X-ray crystallography binding sites were best fitted by assuming cardiolipin interactions comprised two populations: fast interactions and slow interactions (see Methods), and calculating their residence times separately.


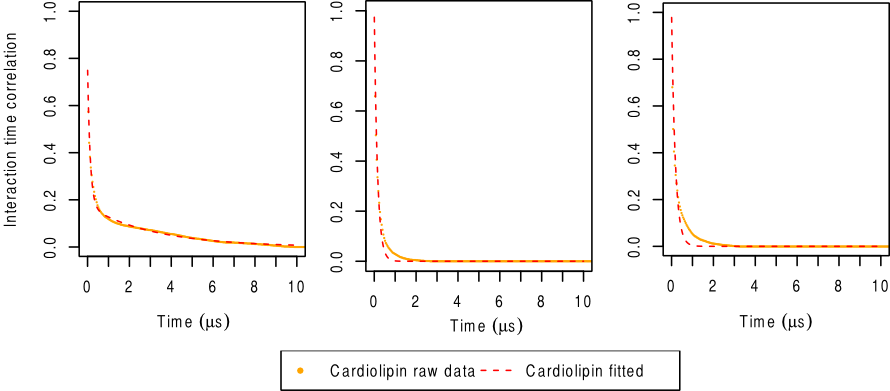


**Fig. S13.** Cardiolipin residence time-correlation functions for the time span of binding between lipids and different classes of binding sites on bovine AAC1. Time-correlation curves derived from simulation data of each AAC simulation, along with their best fit exponential decay curves (dashed lines), are shown for each class of binding site: crystal structure binding sites (left); matrix helix binding sites (middle); and outer leaflet interaction sites (right). The fitted graphs were used to obtain the residence times shown in Table 2 and Fig. S10E.

**Table S1.** Comparison of residues identified as being in the crystal structure binding sites in this study and in a previous simulation of AAC with cardiolipin [34]. Residues in bold are identified in both studies.

| Interaction site name | Residues in crystal structure interaction sites. | |
| --- | --- | --- |
|  | In this study | In Hedger *et al.* [34] |
| CDL800; site I | L67, **S68**, R71, R151, **T154**, **G155** | **S68**, G72, **T154**, **G155** |
| CDL 801; site II | K51, **G52**, G265, K267, K271, **W274** | **G52**, I53, G272, **W274** |
| CDL 802; site III | R170, Y173, **Q174**, **G175**, F176, N177, **T253**, V254 | **Q174**, **G175**, G252, **T253** |
